# Copper treatment during storage reduces *Phytophthora* and *Halophytophthora* infection of *Zostera marina* seeds used for restoration

**DOI:** 10.1038/srep43172

**Published:** 2017-02-22

**Authors:** Laura L. Govers, Els M. van der Zee, Johan P. Meffert, Patricia C. J. van Rijswick, Willem A. Man in ‘t Veld, Jannes H. T. Heusinkveld, Tjisse van der Heide

**Affiliations:** 1 Department of Aquatic Ecology and Environmental Biology, Institute for Water and Wetland research (IWWR), Radboud University, Heyendaalseweg 135, 6525 AJ Nijmegen, The Netherlands; 2 Conservation Ecology Group, Groningen Institute for Evolutionary Life Sciences, University of Groningen (GELIFES), Post Office Box 11103, 9700 CC The Netherlands; 3 Altenburg & Wymenga Ecological Consultants, Suderwei 2, 9269 TZ Veenwouden, The Netherlands; 4 Department of Mycology, National Plant Protection Organisation (NPPO-NL), Post Office Box 9102, 6700 HC Wageningen, The Netherlands; 5 The Fieldwork Company, Stockholmstraat 2B, 9723 BC Groningen, The Netherlands

## Abstract

Restoration is increasingly considered an essential tool to halt and reverse the rapid decline of vital coastal ecosystems dominated by habitat-forming foundation species such as seagrasses. However, two recently discovered pathogens of marine plants, *Phytophthora gemini* and *Halophytophthora* sp. Zostera, can seriously hamper restoration efforts by dramatically reducing seed germination. Here, we report on a novel method that strongly reduces *Phytophthora* and *Halophytophthora* infection of eelgrass (*Zostera marina*) seeds. Seeds were stored in seawater with three different copper sulphate concentrations (0.0, 0.2, 2.0 ppm) crossed with three salinities (0.5, 10.0, 25.0 ppt). Next to reducing seed germination, infection significantly affected cotyledon colour: 90% of the germinated infected seeds displayed a brown cotyledon upon germination that did not continue development into the seedling stage, in contrast to only 13% of the germinated non-infected seeds. Copper successfully reduced infection up to 86% and the 0.2 ppm copper sulphate treatment was just as successful as the 2.0 ppm treatment. Infection was completely eliminated at low salinities, but green seed germination was also dramatically lowered by 10 times. We conclude that copper sulphate treatment is a suitable treatment for disinfecting *Phytophthora* or *Halophytophthora* infected eelgrass seeds, thereby potentially enhancing seed-based restoration success.

Seagrasses form vital habitats in coastal zones across the world and provide important ecosystem services such as coastal protection, carbon sequestration and biodiversity enhancement^1^,^2^,^3^. However, seagrass beds are currently experiencing rapid losses worldwide as a result of human-induced eutrophication, coastal pollution and habitat destruction^4^,^5^. To halt and reverse these losses, many restoration efforts are being undertaken^6^,^7^, with fifty percent of these restoration attempts concerning *Zostera marina* (eelgrass), the most widespread seagrass species in the temperate northern hemisphere^6^. Restoration projects involving eelgrass beds use a variety of techniques including transplantation of sods, rhizome fragments, seeds, seed-bearing shoots, or seedlings. Methods vary among sites, but there has been a general increase of projects using seeds for restoration^8^,^9^,^10^,^11^,^12^,^13^,^14^,^15^. Compared to more traditional approaches, using seeds is more cost-effective^16^, allows for maintaining high genetic diversity of the restored population^17^,^18^, and storage of seeds outside the growing season allows for reduction of seed loss compared to natural conditions^19^.

Although application of seeds in restoration is promising, Govers, *et al*.^20^ recently discovered that two oomycete pathogens, *Phytophthora* sp. and *Halophytophthora* sp. may pose a threat to seed-based restoration as they (1) reduce seed germination by six times, and (2) occur widespread across the Atlantic with very high infection rates (up to 99%). Moreover, the threat for restoration was further highlighted by the finding of *Phytophthora* infection in *Z. marina* seeds of currently ongoing restoration projects in the Netherlands, Denmark, Sweden and the USA^20^.

*Phytophthoras* are well known from terrestrial systems. For instance, the infamous *Phytophthora infestans* causes billions of euros of yearly damage to potato crops in the European union^21^, and related species, *Phytophthora ramorum* and *Phytophthora cinnamomi*, are threatening fragile ecosystems, causing massive die-offs of forests in California and Australia^22^,^23^. Since we now know that *Phytophthora* species can also infect and affect marine plants^20^,^24^, with infection rates as high as 99% in natural populations and a widespread distribution^20^, it is very likely that these pathogens can negatively affect seed-based eelgrass restoration projects. For example, seed loss due to *Phytophthora* and *Halophytophthora* infection can be as high as 40%^20^, which can have serious economic consequences as restoration is very costly with an estimated average price per seed of $0.17^16^.

We therefore aimed to develop a method that reduces seed loss by *Phytophthora* and/or *Halophytophthora* infection during seed storage. We conducted a full-factorial experiment treating stored seeds with three salinities (0.5, 10.0, 25.0 ppt) and three copper sulphate concentrations (0.0, 0.2, 2.0 ppm). Copper-containing compounds are known as effective fungicides counteracting *Phytophthora* infection of agricultural crops^25^,^26^. In addition, low salinity treatment may be a cost-effective and natural method of reducing *Phytophthora* infection, as shown for terrestrial crops due to enhanced susceptibility of salt-stressed plants and increased *Phytophthora* sporangium production in high salinity conditions^27^,^28^,^29^.

## Results

### Seed quality tests prior to the experiment

**Figure 1:**
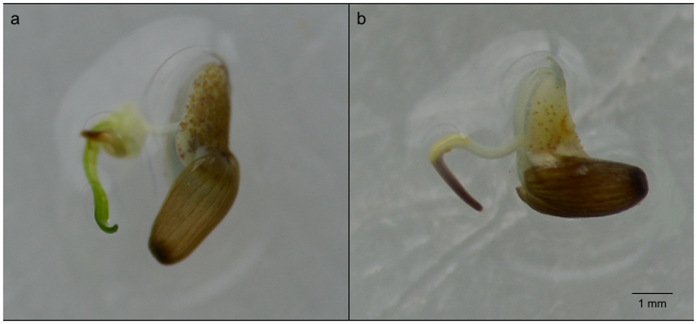
Effect of *Phytophthora* infection on germination colouration of cotyledons. Germinated seeds of *Z. marina* with (**a**) green and (**b**) brown cotyledons. Infected seeds displayed mostly brown cotyledons upon germination.

**Figure 2:**
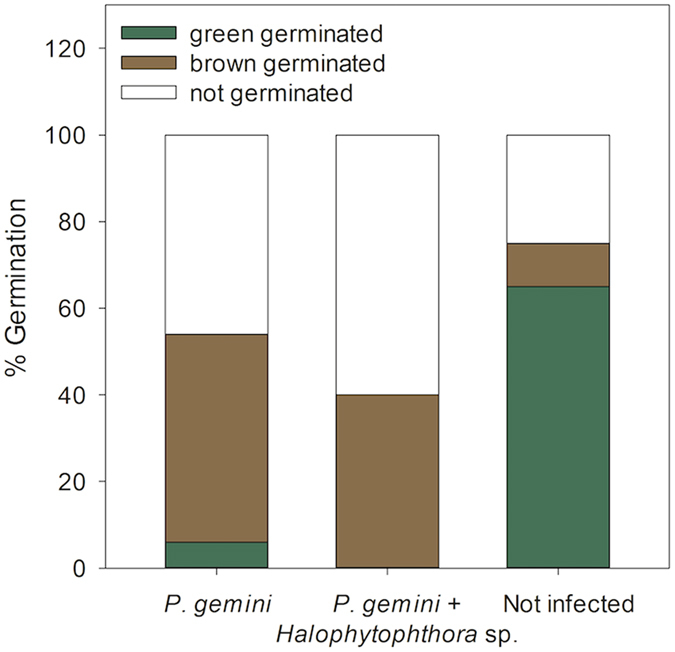
Germination percentages of *P. gemini,P. gemini* + *Halophytophthora* sp. Zostera, and non-infected *Zostera marina* seeds prior to the experiment. Infected seeds germinated mostly with brown cotyledons, whereas non-infected seeds germinated green (n = 102, n = 15, n = 20 for *P. gemini,P. gemini* + *Halophytophthora* sp. Zostera and non-infected seeds respectively). *P. gemini* and *P. gemini* + *Halophytophthora* sp. Zostera infected seeds had significantly more brown-coloured cotyledons than non-infected seeds (X^2^-test, *p* < 0.001 for each infection).

Eighty-six percent of all seeds used in the experiment was infected by either *P. gemini* (74%) or both *P. gemini* and *Halophytophthora* sp. Zostera (11%). None of the seeds were infected by *Halophytophthora* sp. Zostera alone. Germination tests showed that seeds displayed cotyledons of either brown or green colouration upon germination (Fig. 1). Germinated seeds with brown cotyledons were observed not to develop further into seedlings, whereas germinated seeds with green cotyledons did continue to develop. Infection affected colouration of cotyledons of germinated seeds: *P. gemini* and *P. gemini* + *Halophytophthora* sp. Zostera infected seeds had significantly more brown-coloured cotyledons than non-infected seeds (X^2^-test, *p* < 0.001 for each infection) (Fig. 2). Of all *P. gemini* infected seeds, 48% of the seeds had brown-coloured cotyledons after germination, while only 6% had green coloured cotyledons (ratio 8:1). None of the seeds that were infected by both oomycetes displayed green cotyledons after germination. In contrast, only 10% of all non-infected seeds germinated with brown cotyledons and a majority of 65% with green cotyledons (ratio 1:7). Total infection significantly reduced green-coloured germination by 13× (GLMM, z = −5.525, *p* < *0.001*).

### Experimental results

**Figure 3:**
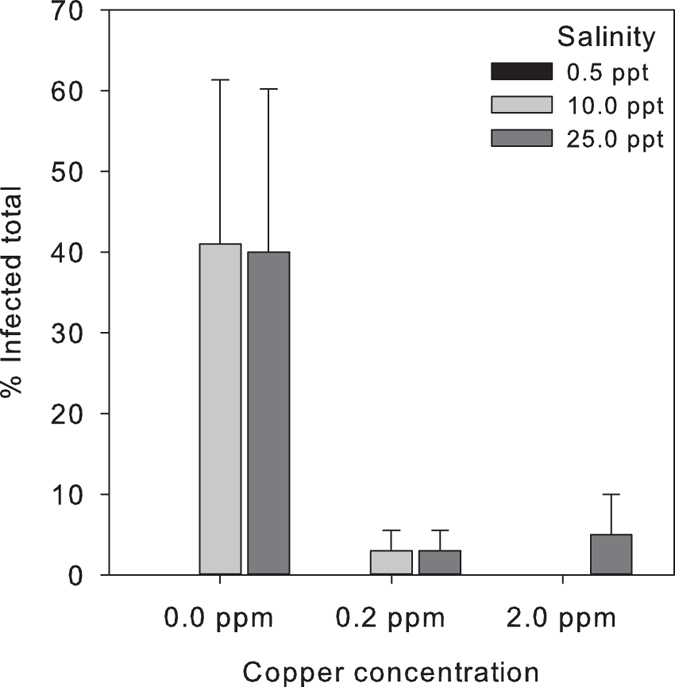
Average percentages of total infected *Zostera marina* seeds per treatment based on individual morphological seed assessment on ParpH. Total infection was significantly reduced by copper treatment (GLMM, z = −2.021, *p* = *0.043*) and by low salinity (GLMM, z = 2.208, *p* = *0.027*). Bars represent sem (n = 4).

In total, only 11% of all seeds was infected after the experiment, 6% by *Halophytophthora* sp. Zostera, 4% by *P. gemini,* and 1% by both *P. gemini* and *Halophytophthora* sp. Zostera. Total infection was significantly reduced by copper treatment (GLMM, z = −2.021, *p* = *0.043*), by 9× in the 0.2 ppm copper treatment and by 6× in the 2.0 ppm copper treatment, with no significant difference between both copper treatments (Fig. 3). Thus, the 0.2 ppm copper sulphate treatment was just as effective reducing infection as the 2.0 ppm copper sulphate treatment. Of the non-copper treated seeds, 36% remained infected at the end of the experiment, implying a reduction in infection of 2.5× during storage without treatment. Low salinity (0.5 ppt) completely eliminated infection (GLMM, z = 2.208, *p* = *0.027*): none of the 0.5 ppt treated seeds were infected by *P. gemini* or *Halophytophthora* sp. Zostera after the experiment. The 10.0 and 25.0 ppt salinity treatments had similar (14 and 16% respectively) infection rates (Fig. 3a).

**Figure 4:**
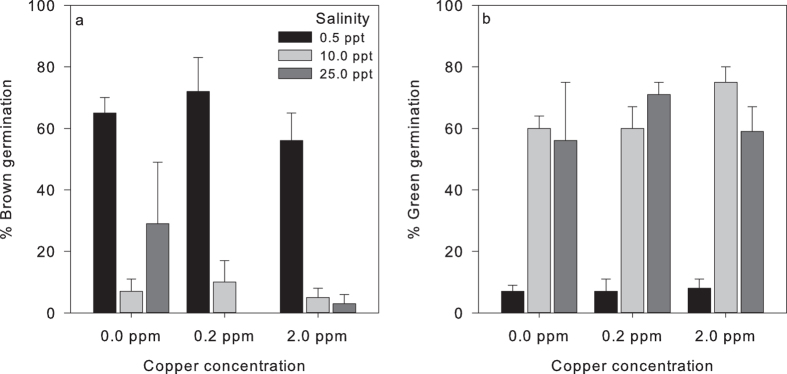
Average percentages of *Zostera marina* seed germination per treatment. (**a**) Treatment effects on Green seed germination and (**b**) treatment effects on brown seed germination. Infection significantly reduced germination (GLMM, z = −2.948, *p* = *0.0032*) and infected seeds displayed more brown cotyledons than non-infected seeds (GLMM, z = −3.975, *p* < *0.001*). Seeds stored in freshwater displayed significantly reduced germination (GLMM, z = 5.473, *p* < 0.001) and higher brown cotyledon levels upon germination (GLMM, z = −5.189, *p* < *0.001*). Bars represent sem (n = 4).

Infection significantly reduced *Z. marina* green seed germination by 1.5× (Fig. 4a): 31% of total infected seeds showed green germination versus 46% of the non-infected seeds (GLMM, z = −2.948, *p* = *0.0032*). In contrast, more infected seeds germinated displayed brown cotyledons (39%) than non-infected seeds (26%) (GLMM, z = −3.975, *p* < *0.001*) (Fig. 4b). In addition to infection, also salinity strongly affected germination. Seeds stored in freshwater (0.5 ppt) displayed significantly reduced green germination (Fig. 4a), <7.5% germinated with green cotyledons in contrast to >55% green germination of seeds stored at higher salinities (GLMM, z = 5.473, *p* < 0.001). Conversely, freshwater storage promoted brown seed germination: >50% of the 0.5 ppt stored seeds displayed brown germination versus only 7–30% of seeds stored at higher salinities (GLMM, z = −5.189, *p* < *0.001*). Copper treatment affected neither green nor brown germination.

## Discussion

Restoration of eelgrass beds using seeds often suffers from low recruitment rates^30^,^31^,^32^,^33^. Large numbers of seed may be lost due to predation^15^,^34^, bioturbation^35^ and currents and waves^36^. In addition, the recently discovered *Phytophthora* and *Halophytophthora* species^20^ may also enhance seed loss owing to strongly reduced germination of infected seeds. Here, we present a successful, inexpensive, and simple method to strongly reduce infection (by 86%) in seeds stored for restoration purposes, using low concentrations of copper sulphate dissolved in seawater. Moreover, we also report on the first successful application of a fungicide on a *Halophytophthora* species. This has never been attempted before because these oomycetes, although related to *Phytophthora* species, are generally saprophytes, decomposing leaves in salt marshes and mangrove forests^37^,^38^,^39^.

The use of copper as a fungicide already started in 19^th^ century France^40^. Although not known at the time, copper-based compounds are effective fungicides because they are lethal to sporangia, zoospores and chlamydospores of fungi, algae and oomycetes^25^,^26^. Nowadays, many copper-based chemicals are registered and commercially used to reduce crop loss from *Phytophthora* infection^41^,^42^,^43^. The most widely used copper-based agrochemicals include copper oxychloride, copper hydroxide and cuprous oxide. These chemicals, however, have low water solubility and are therefore applied in relative large amounts^40^. This may cause severe environmental damage because copper is toxic to most higher plants and animals above generally 0.20–0.30 μg g^−1^ DW^44^. Copper toxicity in plants may lead to lipid peroxidation, protein oxidation and DNA damage^40^. In addition, copper toxicity may induce iron deficiency leading to leaf chlorosis^44^. Thus, a fine balance exists between the use of copper-based chemicals for plant protection and phytotoxicity. Another copper-based compound, copper sulphate (CuSO_4_), often used in an aqueous medium as algicide in for instance ponds, fish tanks, and swimming pools^25^,^45^,^46^, has also been effectively used to eliminate *Phytophthora* propagules from natural streams^25^, and both copper and sulphate ions are naturally present in ecosystems. We therefore selected this fungicide/algicide for the potential treatment of infected *Z. marina* seeds stored for restoration purposes. Seed treatment with copper was successful and concentrations of 0.2 ppm were just as effective in reducing infection as 2.0 ppm treatments. Similar disease reduction was also found in *Phytophthora cryptogea* infected gerberas treated with a 0.28 ppm copper solution^47^. Both copper concentrations used in our experiment (0.2 ppm and 2.0 ppm) are most likely below copper toxicity levels for *Z. marina* because seed germination was not affected by these treatments. This is surprising, because Lyngby and Brix^48^ showed that copper concentrations of 5 μM (0.32 ppm) or more can be toxic to adult *Z. marina* plants by reducing growth and inducing leaf necrosis. Possibly, high copper concentrations are more detrimental to adult plants than to seeds. Nevertheless, we recommend a 0.2 ppm instead of a 2.0 ppm copper treatment to reduce *Phytophthora* and *Halophytophthora* infection of *Z. marina* seeds. In addition, we have used these copper treatments in containers where concentrations could be carefully controlled. We do not recommend application in the field as one would need to account for currents and tides and effective concentrations would likely only to be reached when applying large amounts of copper sulphate, potentially leading to environmental damage. Thus, seed treatment by copper sulphate is recommended only during storage for restoration purposes and not in a natural population in the field.

Results of salinity treatments on infection and seed germination of *Z. marina* seeds were mixed; although infection by *Phytophthora* and *Halophytophthora* was completely eliminated in fresh water (0.5 ppt), the quality of the germinated seeds (brown instead of green cotyledons) was also severely impaired. Reduction of infection by low salinities may be due to limited tolerance of halophile *P. gemini* and *Halophytophthora* sp. Zostera. However, preliminary tests showed growth of these oomycetes on a selective growth medium (ParpH) with sterilized freshwater (Meffert & Van Rijswick, *pers. comm. 2016*). In addition, *P. inundata*, a close relative of *P. gemini*, is known from both freshwater and marine environments^24^, indicating a broad salt tolerance. Therefore, we conclude it is more likely that *P. gemini* and *Halophytophthora* sp. Zostera were outcompeted by other microorganisms (fungi or algae) in freshwater, also because we observed a black fungus (*Phialophora malorum*) on the seed coats in the 0.5 ppt treatments (including 0.2 and 2.0 ppm copper treatments). *P. malorum* was only observed in the freshwater treatments and may not only have affected *Phytophthora* or *Halophytophthora* infection by competition, but potentially also reduced seed germination in those treatments. This, however, needs further investigation.

Our results are in line with the effects of salinity on *Phytophthora* infection in terrestrial crops. In those systems, increased salinity generally enhances the effects of *Phytophthora* infection^27^,^28^,^29^. This is due to enhanced susceptibility to pathogens as a result of salt-stress^29^, but also due to increased sporangium production of *Phytophthora*^49^ under saline conditions. A similar trend – lower *P. gemini* and *Halophytophthora* sp. Zostera infection at low salinities – indicates adaptation of these species to the marine environment^50^. Correspondingly, another oomycete pathogen adapted to saline conditions, *Labyrinthula zosterae* (wasting disease), is also inhibited by low salinities^51^,^52^. In addition, *Z. marina* appears more susceptible to *Labyrinthula* infection at high salinities^53^,^54^.

Reduced quality of the pre-seedling stage of freshwater-treated seeds indicates that storing seeds at very low salinities for a longer period (110 days in our case) is detrimental to seed quality. These results are in concert with the findings of Xu, *et al*.^11^ who reported severely impaired morphology and growth of *Z. marina* seedlings grown at salinities <20 ppt. Although germination tests were conducted at 20.0 ppt, negative effects of storing seeds in freshwater appeared to be lasting. In addition, storing *Zostera* seeds at low salinities may also increase seed loss by enhancing premature germination^15^,^55^,^56^. Taking this into account and because both infection and germination were similar in the 10.0 and 25.0 ppt salinity treatments, we recommend storing seeds for restoration purposes at higher (i.e. >20 ppt) salinities.

To conclude, we present a novel method to reduce *Z. marina* seed loss by *Phytophthora* and *Halophytophthora* infection in seeds to be used for restoration purposes.

Treatment with 0.2 ppm copper sulphate strongly reduced infection but did not affect seed quality and is therefore recommended during storage of infected seeds. Although storing seeds at low salinity (0.5 ppt) effectively reduced oomycete infection, storage under these conditions is not recommended since this treatment strongly impaired seed quality. Infection during storage has been documented from multiple countries (the Netherlands, Denmark, Sweden, USA) and seed loss due to infection may be as high as 40%^20^. These results may thus be applicable to all seed-based *Z. marina* restoration projects around the world^8^,^9^,^11^,^15^,^55^,^57^

## Materials and Methods

### Seed collection

Seed-bearing shoots where collected at low tide, in an intertidal mixed *Zostera* marina/*Zostera noltii* meadow at the German barrier island Sylt (54.799 °N, 8.296°E). Collection took place in early September 2015 and shoots were transported to the lab at 6 °C. Here, these shoots where placed in aerated water tanks (15 kg shoots per 10,000 L, 30 ppt salinity, ambient temperature and light conditions, closed circulation), allowing the seeds to further ripen. Seeds were collected from the bottom of the aerated water tanks after 3 weeks. Of these, we selected 360 seeds of medium to advanced maturity^11^ for the experiment. Another 30 seeds from 4 different storage containers (120 in total) were individually tested for *P. gemini* and/or *Halophytophthora* sp. Zostera infection before the start of the experiment using both visual and molecular techniques^20^.

### Experimental set-up

To study the effects of both copper sulphate and salinity on *Phytophthora a*nd *Halophytophthora* infection of *Z. marina* seeds, we crossed three copper sulphate (CuSO_4_) treatments (0.0, 0.2, 2.0 ppm) with three salinity treatments (0.5, 10.0, 25.0 ppt), resulting in 9 different treatments. Copper sulphate concentrations were based upon minimal algicidal levels (0.2 ppm)^58^ and maximal copper concentrations (~2.0 ppm) advised for usage to control fungi in ponds and fish tanks with high alkalinity^45^. We used 4 replicates per treatment, using 0.47 L cylindrical pvc units that were closed at the bottom^59^. Each unit was equipped with a porous membrane in the lower half of the column, through which individual aeration was added to ensure total oxygenation of the water column. Through the same membrane, each experimental unit was connected to a peristaltic pump that continuously injected water from a basin with the assigned treatment, into each unit. Experimental units where placed in a large, empty tank, allowing excess water to overflow, thus creating a continuous flow-through system^60^,^61^,^62^ (Masterflex ® 7568–10 Peristaltic Tubing Pumps, Cole-Parmer, USA) with a refreshment rate of once every two days (10 ml h^−1^). Overflow water was discharged to a waste water container to ensure that water of the experimental units was never brought in contact with that of other units. Salinity treatments were prepared from deionized water and Tropic Marin synthetic sea salt^63^ and checked twice a week using a multi-probe meter (556 MPS, YSI). Copper sulphate (CuSO_4_) was added to the treatment water basin and measured every week using a Copper checker (Hanna, HC – HI702) and adjusted if necessary. At the start of the experiment, Copper concentrations decreased rapidly as a result of adsorption of the copper ions to the treatment basins, thus requiring regular corrections by addition of CuSO_4_. However, after an initial charging phase of two weeks, copper concentrations remained constant. We placed 10 seeds in a nylon mesh bag (0.1 mm) attached to a glass bead anchor in the middle of every experimental unit at the start of the experiment. The entire experiment was conducted in a dark, climate controlled room at 5 °C to ensure optimal seed storage conditions^8^,^9^,^55^,^57^ and to prevent premature germination^15^.

### Phytophthora and Halophytophthora analysis

After 105 days of incubation, the experiment was terminated and seeds were retrieved from the mesh bags and stored in 20 ml vials with 5 ml water from the assigned treatment. These samples, 360 seeds in total, were immediately transferred to the lab for *Phytophthora* and *Halophytophthora* analysis. Next, seeds were individually placed (one seed per well) on sterile 12 wells tissue culture plates with a growth area of 3.8 cm^2^ per well with ParpH selective growth medium^64^, see Govers, *et al*.^20^ for a visual representation of this method. ParpH is an oomycete-selective growth agar growth medium to which selected antibiotics are added to promote growth of *PhytophthoraHalophytophthora*, and *Pythium* species and to suppress growth of non-pythiaceous fungi^64^. Seeds were incubated on ParpH for 4 weeks, with a natural daylight cycle at room temperature (18–20 °C) with 2–3 ml of artificial seawater added to every well. After 3 and 7 days, presence of *P. gemini* and/or *Halophytophthora* sp. Zostera was individually scored for each seed by identification based on colony morphology^20^. In addition, seed germination and cotyledon colour (brown/green) of seeds incubated on ParpH with seawater was score daily up to four weeks of seed incubation. Oomycete species identification was also performed by sequence analysis of the internal transcribed spacer regions (ITS1 and ITS2) of the ribosomal DNA gene repeat on 4 samples of colonies additionally grown on cherry decoction agar (CHA)^24^. See Govers, *et al*.^20^ for more details on the visual and molecular identification methods.

### Statistical analysis

Infection (based on visual assessment on parpH) and germination were analyzed by Generalized linear mixed models with binomial distribution (GLMM, lme4 package in R 3.2.3). We included treatments (salinity, copper addition) as fixed factors and experimental unit as random factors in our models. First, we tested the complete model with all treatments and interactions and then stepwise reduced the model by excluding non-significant interactions, starting with the most complex interactions. We hereby reduced the statistical model until only significant factors were left^65^. We used Tukey HSD posthoc tests to separate treatment effects. We report GLMM results as z values and P values. Effects of infection on cotyledon colouration were additionally tested by Chi-squared tests. P values < 0.05 were considered statistically significant.

## Additional Information

**How to cite this article:** Govers, L. L. *et al*. Copper treatment during storage reduces **Phytophthora** and *Halophytophthora* infection of *Zostera marina* seeds used for restoration. *Sci. Rep.* **7**, 43172; doi: 10.1038/srep43172 (2017).

**Publisher's note:** Springer Nature remains neutral with regard to jurisdictional claims in published maps and institutional affiliations.
